# Activation of hedgehog signaling is not a frequent event in ovarian cancers

**DOI:** 10.1186/1476-4598-8-112

**Published:** 2009-11-27

**Authors:** Ling Yang, Jing He, Shuhong Huang, Xiaoli Zhang, Yuehong Bian, Nonggao He, Hongwei Zhang, Jingwu Xie

**Affiliations:** 1 Institute of Developmental Biology, School of Life Sciences, Shandong University, Jinan 250100, PR China; 2 Sealy Center for Cancer Cell Biology, Department of Pharmacology and Toxicology, University of Texas Medical Branch at Galveston, Texas 77555-1048, USA; 3 Wells Center for Pediatric Research, Department of Pediatrics, Division of Hematology and Oncology, Indiana University Simon Cancer Center, Indiana University School of Medicine, Indiana 46202, USA

## Abstract

The hedgehog (Hh) signaling pathway regulates many processes of development and tissue homeostasis. Activation of hedgehog signaling has been reported in about 30% of human cancer including ovarian cancer. Inhibition of hedgehog signaling has been pursued as an effective strategy for cancer treatment including an ongoing phase II clinical trial in ovarian cancer. However, the rate of hedgehog signaling activation in ovarian cancer was reported differently by different groups. To predict the successful for future clinical trials of hedgehog signaling inhibitors in ovarian cancer, we assessed hedgehog pathway activation in 34 ovarian epithelial tumor specimens through analyses of target gene expression by *in-situ *hybridization, immunohistochemistry, RT-PCR and real-time PCR. In contrast to previous reports, we only detected a small proportion of ovarian cancers with hedgehog target gene expression, suggesting that identification of the tumors with activated hedgehog signaling activation will facilitate chemotherapy with hedgehog signaling inhibitors.

## Findings

Ovarian cancer is the most deadly type of gynecological cancers. Epithelial ovarian cancer is the major ovarian malignancy consisting of five histological subtypes: serous, mucinous, endometrioid, transitional and clear cell [1,2]. Overall, the 5-year relative survival of ovarian cancer patients is 46%. If diagnosed at the localized stage, the 5-year survival rate is 93% [3]. The survival of ovarian carcinoma patients has not improved significantly for years due to lack of knowledge for molecular mechanisms underlying ovarian cancer development. Thus, identifying novel markers for early diagnosis of ovarian cancer can significantly reduce the mortality of ovarian cancer and possibly facilitates targeted cancer therapeutics.

The hedgehog (Hh) signaling pathway regulates many processes of development and tissue homeostasis [4,5]. In the absence of the ligand Hh, hedgehog receptor (PTCH1 or PTCH2) inhibits smoothened (SMO) signaling. When Hh binds to PTCH1, SMO is able to signal, eventually resulting in formation of activated transcriptional factor Gli (Gli1 and Gli2) molecules and elevated expression of the target genes (e.g. PTCH1, Gli1, HIP etc). Activation of hedgehog signaling has been reported in about 30% of human cancer [6] including ovarian cancer. Inhibition of hedgehog signaling has been pursued as an effective strategy for cancer treatment including an ongoing clinical trial in solid tumors [7] such as ovarian cancer. Previous studies showed different results of hedgehog signaling activation in ovarian cancer. While one study suggests activation of hedgehog signaling in virtually all tumors examined using mainly immunohistochemistry [8], another study indicated a much low rate of hedgehog signaling activation [9]. It appears that a comprehensive study on ovarian cancer hedgehog signaling is necessary in order to predict the feasibility of clinical trials of hedgehog signaling inhibitors in ovarian cancer. In this study, we examined hedgehog pathway activation in 34 ovarian cancer specimens through analyses of target gene expression by *in-situ *hybridization, immunohistochemistry, RT-PCR and real-time PCR (Additional file 1).

**Figure 1:**
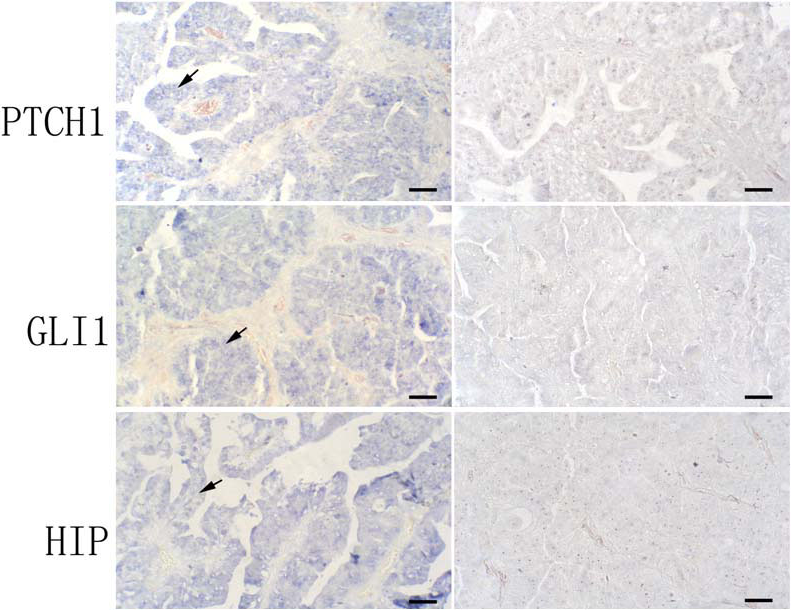
**Expression of *PTCH1, GLI1 *and *HIP1 *in ovarian cancer**. *PTCH1, GLI1 *and *HIP *transcript (blue as positive) was detected by *in situ *hybridization in a well-differentiated serous papillary adenocarcinoma (the left panel), and the right panel pictures are their controls with respective sense probes (Bars indicate 50 μm).

**Figure 2:**
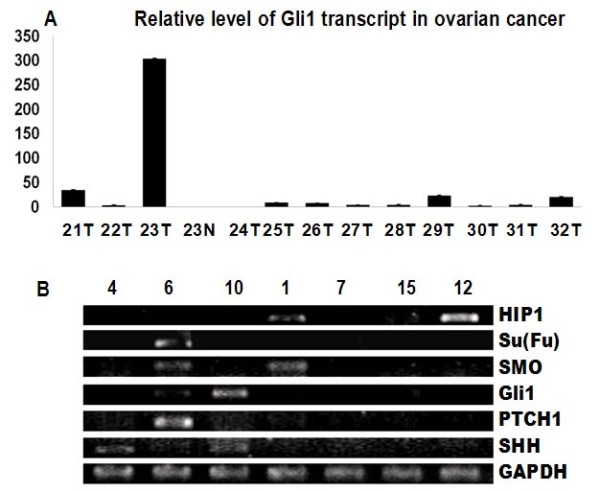
**PCR detection of hedgehog signaling in ovarian cancer specimens**. **A**. Real-time PCR analysis of *GLI1 *expression in ovarian cancer was performed as described in Materials and Methods. *Gli1 *transcript was shown here. **B**. PCR detection of *SHH, PTCH1, GLI1, SMO, HIP1 *and *Su(Fu) *transcripts in ovarian cancers. GAPDH is the endogenous control. Numbers listed indicate specimen number.

**Figure 3:**
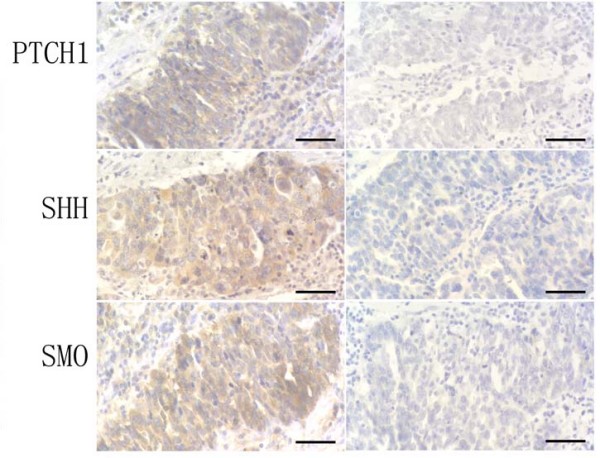
**Expression of PTCH1, SHH and SMO protein in ovarian cancer**. PTCH1, SHH and SMO protein (yellow as positive) was detected by immunohistochemistry in a poorly-differentiated serous papillary adenocarcinoma (left panel), and the right panel pictures are controls without primary antibody (Bars indicate 50 μm).

Previous studies indicated that sonic hedgehog expression is elevated in ovarian cancer [1,8-10]. These and other studies led to a phase II clinical studies in ovarian cancer using hedgehog inhibitors. We examined three hedgehog target genes in these specimens: *PTCH1, Gli1, HIP1*. Tumors with expression of two hedgehog target genes are regarded as hedgehog signaling activated tumors. *PTCH1 *expression was detected in 9 of 34 (~26%). In *in-situ *hybridization analysis, most positive staining of *PTCH1 *was seen in the cancer tissues (Figure 1 indicated by arrows) not in the adjacent stroma. The antisense probe of *PTCH1 *gave positive (blue) signal but the sense probes did not show staining, indicating the specificity of *in-situ *hybridization. Further analysis did not reveal association between *PTCH1 *expression and tumor subtypes, stage or other characteristics (Additional file 2). The result of *in-situ *hybridization was confirmed in tumor specimens with 70% of tissue mass by PCR amplification (Figure 2). Expression of PTCH1 protein (Figure 3) was further confirmed by immunohistochemistry in the specimens with elevated expression of *PTCH1 *transcript.

*GLI1 and HIP1 *expression was detected in 9 of 34 (~26%) and 7 of 34 (~21%) cancers respectively. Like *PTCH1*, most positive staining of *GLI1 and HIP1 *was shown in cancer (Fig. 1 indicated by arrows), not in stromal tissues. Real-time PCR confirmed the results from *in-situ *hybridization showed that normal tissues had low or no level of *GLI1 *expression whereas tumor tissues had elevated levels of *GLI1 *(Figure 2). Like *PTCH1*, we did not find association of *Gli1 *expression (or *HIP1*) with any tumor characteristics. The result of RT-PCR shown in Figure 2 was consistent with result of *in-situ *hybridization. Further analysis indicated that *PTCH1 *co-expressed with *GLI1 *(p = 0.0033), but not with *HIP1 *expression. *HIP1 *expression was only detected in 2 specimens which expressed *PTCH1 *or *Gli1*, indicating that *HIP1 *is not highly expressed in ovarian tissues or is silenced in cancers as reported in other studies [11,12]. Only 7 out of 34 ovarian cancer specimens (~21%) have elevated expression of two hedgehog target genes (Additional file 2), indicating that hedgehog signaling activation is not very common in ovarian cancer.

**Figure 4:**
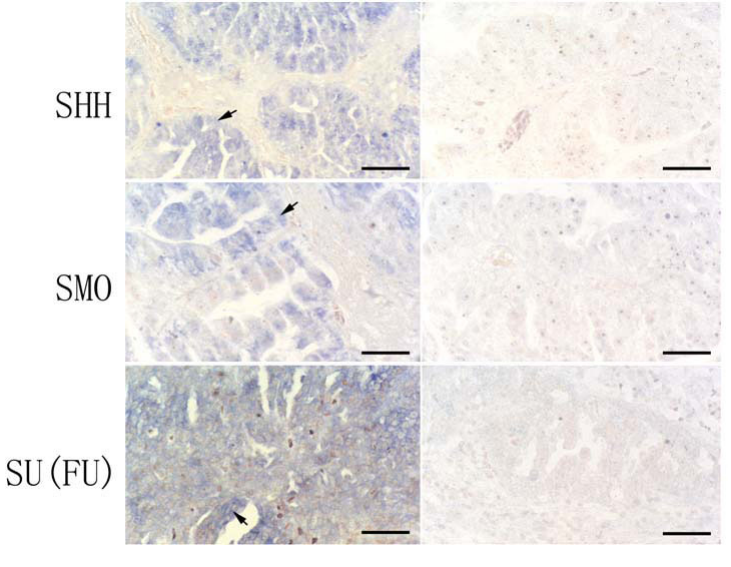
**Expression of *SHH*, *SMO *and *Su(Fu) *in ovarian cancer**. *SHH*, *SMO *and *Su(Fu) *transcript (blue as positive) was detected by *in situ *hybridization. The positive stain of *SHH*, *SMO *was shown in a well-differentiated serous papillary adenocarcinoma and the positive stain of *Su(Fu) *was shown in a poorly-differentiated serous papillary adenocarcinoma (left panel), and the right panel pictures are controls of *in situ *hybridization with respective sense probe (the bar indicates 50 μm).

To understand the molecular basis of hedgehog signaling activation in ovarian cancer specimens, we assessed expression of hedgehog signaling components in ovarian cancer. Expression of *SHH *was found in 11 of 34 (~32%) ovarian cancers (Additional file 2). By *in-situ *hybridization, we found *SHH *expression mainly in cancer tissues (Figure 4 indicated by arrows). PCR also showed that normal tissues had low or no level of *SHH *expression, while tumor tissues had elevated RNA level of *SHH *(Figure 2). Further analysis revealed that *SHH *did not co-express with hedgehog target genes *PTCH1*, *GLI1 or HIP*. *SHH *expression was not associated with expression of hedgehog target genes (Additional file 2), indicating that SHH expression alone is not responsible for Hh pathway signaling activation in ovarian cancer.

In addition to SHH, we also detected expression of *SMO *and *Su(Fu) *in 17 ovarian cancers. Although we found SMO expression in 4 (~24%), and Su(Fu) in 4 (~24%) tumors respectively. Most tumors with elevated expression of *Gli1 *and *PTCH1 *had no expression of SMO, suggesting that SMO expression was not responsible for hedgehog signaling activation in ovarian cancer.

The result of *in situ *hybridization was confirmed by semi-quantified RT-PCR showing in Figure 2. Expression of SHH and SMO protein (Figure 3) was also detected by immunohistochemistry in the specimens with expression of *SHH *and *SMO *by *in-situ *hybridization and RT-PCR. In summary of our data, we found only a small proportion of ovarian cancer specimens with elevated hedgehog signaling activation. Our data also showed that elevated SHH expression is not always associated with Hh target genes expression. Based on our data, we caution that clinical trials with hedgehog signaling inhibitors in ovarian cancer may be successful in selected patient population with elevated Hh target genes.

Using several approaches, we showed that the percentage of hedgehog signaling activation is low in ovarian cancer. We only found 20% of tumor specimens with detectable expression of two hedgehog target genes, as indicated in real-time PCR and *in-situ *hybridization analyses. Our data suggest that it is necessary to identify the right population of ovarian cancer patients in the clinical trials with hedgehog signaling inhibitors. In addition, we found that even in the tumor with elevated expression of hedgehog target genes *Gli1 *and *PTCH1*, expression of *SHH *is not necessarily high, suggesting other mechanisms of hedgehog signaling activation in the cancer. The fact that hedgehog signaling activation is not associated with any particular subtypes of ovarian cancer suggests that the morphological classification of ovarian cancer may not reflect the molecular pathogenesis of this disease. It will be of interests to establish an animal model to study hedgehog signaling-mediated carcinogenesis in ovary.

Different results have been reported on hedgehog signaling activation in ovarian cancer [8,9]. There are several reasons for this discrepancy. First, different standards have been used to define Hh signaling activation in ovarian cancer. One group used immunohistochemistry to detect expression Hh targets PTCH1 and Gli1 in the tumor specimens [8] whereas we and others assessed the expression of Hh target genes by several methods: *in-situ *hybridization, real-time PCR and immunohistochemistry. Although it is possible that our results may under estimate the frequency of hedgehog signaling, we predict that screening of Hh signaling activated tumors will significantly improve the successful rate of clinical trials using Hh signaling inhibitors. It is also possible that the involvement of Hh signaling in human cancers may be context dependent, occurring in some tissues or cell lines but not in others. Evidence suggests that Hh signaling may be involved in maintaining cancer stem cell proliferation [13,14].

Taken together, our findings suggest that activation of the Hh pathway is not frequent in ovarian cancer. The fact that SHH expression is not correlated with Hh target gene expression suggests that there are other mechanisms responsible for Hh pathway activation. Our studies predict that targeted inhibition of the hedgehog pathway may be only effective in a small percentage of ovarian cancer patients.

## Abbreviations

HIP: hedgehog:interacting protein; Su(Fu): suppressor of fused; PTCH1: human homologue of *patched *1; Shh: sonic hedgehog; SMO: smoothened.

## Competing interests

The authors declare that they have no competing interests.

## Authors' contributions

LY carried out the *in-situ *hybridization and immunohistochemistry, performed the statistical analysis and drafted the manuscript. SH and YB participated the *in-situ *hybridization and immunohistochemistry. JH, XZ and NH performed the real-time PCR analysis of ovarian cancer. JX and HZ designed and planed the experiment, drafted the manuscript. All authors read and approved the final manuscript.

## Authors' Information

Dr JX is a professor at Indiana University Wells Center for Pediatric Research and IU Simon Cancer Center, with a focus on hedgehog signaling in human cancer. He was involved in the initial linking of hedgehog signaling to human cancer in 1996 and was the first to report activated SMO mutations in human cancer. Professor HZ from Shandong University has expertise in developmental biology and interests in linking developmental pathways to human diseases. In the last few years, these two groups have reported links of hedgehog signaling to several cancer types, including liver, esophageal, colon and gastric cancers using comprehensive analyses of hedgehog target gene expression.

## Supplementary Material

Additional file 1 Materials and methods. Click here for file

Additional file 2 Additional table. Click here for file
