# Supplementary material for: Activation of hedgehog signaling is not a frequent event in ovarian cancers
Source: Mol Cancer. 2009 Nov 27;8:112. doi: 10.1186/1476-4598-8-112 (PMC2787497; doi:10.1186/1476-4598-8-112)
Supplement: Additional file 1 — Materials and methods. [file 1476-4598-8-112-S1.DOC]

**MATERIALS AND METHODS**

# Tissue samples

A total of 34 specimens of ovarian tissues were used. Of those, 17 specimens were received as discarded materials from General Surgery of the Shan Dong Qi Lu Hospital, Jinan, China. Pathology reports and H&E stained sections of each specimen were reviewed to determine the nature of the disease and the tumor histology. The remaining specimens were from the University of Texas Medical Branch Tumor Bank with approval from Institutional Research Board. None of the patients had reported chemotherapy or radiation therapy prior to specimen collection.

## ***In-situ* hybridization**

*In-situ* hybridization was performed according to the manufacture’s instructions (Roche Molecular Biochemicals) and our published protocol [1-3]. In brief, tissues were fixed with 4% paraformaldehyde in phosphate buffered saline (PBS) and embedded with paraffin. Then 6 μm thick tissue sections were mounted onto Poly-L-Lysine slides. Samples were treated with proteinase K (20 μg/ml) at 37°C for 15 min, refixed in 4% paraformaldehyde, and hybridized overnight with a digoxigenin-labeled RNA probe (at a final concentration of 1 μg/ml). The hybridized RNA was detected by alkaline phosphatase-conjugated anti-digoxigenin antibodies (Roche Molecular Biochemicals), which catalyzed a color reaction with the substrate NBT/BCIP (Roche Molecular Biochemicals). Blue signal indicated positive hybridization. We regarded tissues without blue signals as negative. As negative controls, sense probes were used in the hybridization and no signals were observed. ***In*** ***situ*** **hybridization**s were repeated at least twice for each tissue sample with similar results.

**Immunohistochemistry** A standard avidin-biotin immunostaining technique was performed using a kit from Vector laboratories using specific antibodies to Su(Fu) (Santa Cruz Biotechnology Cat# 10933), PTCH1 (Santa Cruz Biotechnology Cat# 6149), HIP (R&D systems Cat# AF1568), SMO (ab13118-50, Abcam, Cambridge, UK) and Shh (Santa Cruz Biotechnology Cat# 9024). Positive staining was in red or brown. The specificity of antibodies was tested using the very peptide used for raising the antibodies, which abolished the specific staining [4]. Hematoxylin was used for counter staining (positive as blue in the nucleus).

**RT-PCR and real-time PCR analyses** Total RNA of cells was extracted using a RNA extraction kit from Promega according to the manufacturer (Promega, Madison, WI), and quantitative PCR analyses were performed according to a previously published procedure [1, 2, 5]. Triplicate C_T_ values were analyzed in Microsoft Excel using the comparative C_T_(ΔΔC_T_) method as described by the manufacturer (Applied Biosystems, Foster City, CA). The amount of target (2^-ΔΔCT^) was obtained by normalization to an endogenous reference (18S RNA) and relative to a calibrator. RT-PCR was performed using primers and conditions previously reported [2].

**Statistical analysis** was performed by two tailed Fisher’s exact test. The association of mRNA transcript expression with various clinicopathologic parameters was also analyzed, a *p* value <0.05 was considered to be statistically significant [1, 3].

1. Huang S, He J, Zhang X, Bian X, Yang L, Xie G, Zhang K, Tang W, Stelter AA, Wang Q *et al*: **Activation of the hedgehog pathway in human hepatocellular carcinomas**. *Carcinogenesis* 2006, **27**(7):1334-1340.

2. Ma X, Chen K, Huang S, Zhang X, Adegboyega PA, Evers BM, Zhang H, Xie J: **Frequent activation of the hedgehog pathway in advanced gastric adenocarcinomas**. *Carcinogenesis* 2005, **26**(10):1698-1705.

3. Ma X, Sheng T, Zhang Y, Zhang X, He J, Huang S, Chen K, Sultz J, Adegboyega PA, Zhang H *et al*: **Hedgehog signaling is activated in subsets of esophageal cancers**. *Int J Cancer* 2006, **118**(1):139-148.

4. Sheng T, Li C, Zhang X, Chi S, He N, Chen K, McCormick F, Gatalica Z, Xie J: **Activation of the hedgehog pathway in advanced prostate cancer**. *Mol Cancer* 2004, **3**:29.

5. Athar M, Li C, Tang X, Chi S, Zhang X, Kim AL, Tyring SK, Kopelovich L, Hebert J, Epstein EH, Jr. *et al*: **Inhibition of smoothened signaling prevents ultraviolet B-induced basal cell carcinomas through regulation of Fas expression and apoptosis**. *Cancer Res* 2004, **64**(20):7545-7552.
